# (*E*)-Benz­yl(1-phenyl­ethyl­idene)amine

**DOI:** 10.1107/S1600536813019636

**Published:** 2013-07-20

**Authors:** Sean H. Majer, Joseph M. Tanski

**Affiliations:** a Department of Chemistry, Vassar College, Poughkeepsie, NY 12604, USA

## Abstract

The title compound, C_15_H_15_N, represents an *E* isomer. The mol­ecule exhibits a minor [9.1 (2)%] disorder with methyl­benzyl­idene and benzyl groups inter­changing their positions. The C=N bond length is 1.292 (2) Å. The mol­ecular geometry is essentially planar, with the maximal twist of 8.5 (3)° for the benzyl group. The herringbone packing arrangement does not exhibit any π-stacking inter­actions.

## Related literature

For information on the synthesis of (*E*)-benz­yl(1-phenyl­ethyl­idene)amine, see: Guthrie *et al.* (1973 ▶); Willoughby & Buchwald (1994 ▶). For the crystal structures of similar imines, see: Bruno *et al.* (2012 ▶); Filarowski *et al.* (1999 ▶); Liu *et al.* (1997 ▶).
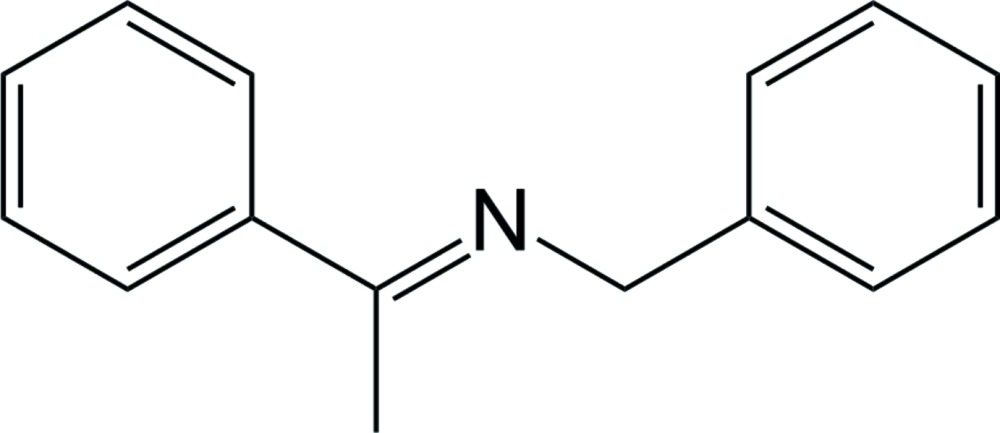


## Experimental

### 

#### Crystal data

C_15_H_15_N
*M* *_r_* = 209.28
Monoclinic, 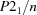

*a* = 5.4633 (4) Å
*b* = 10.4173 (8) Å
*c* = 20.2426 (15) Å
β = 97.308 (1)°
*V* = 1142.71 (15) Å^3^
*Z* = 4
Mo *K*α radiation
μ = 0.07 mm^−1^
*T* = 125 K
0.32 × 0.21 × 0.03 mm

#### Data collection

Bruker APEXII CCD diffractometer
Absorption correction: empirical (using intensity measurements) (*SADABS*; Bruker, 2007 ▶) *T*_min_ = 0.978, *T*_max_ = 0.998
18404 measured reflections
3496 independent reflections
2509 reflections with *I* > 2σ(*I*)
*R*_int_ = 0.042

#### Refinement

*R*[*F* ^2^ > 2σ(*F* ^2^)] = 0.047
*wR*(*F* ^2^) = 0.127
*S* = 1.03
3496 reflections
160 parameters
105 restraints
H-atom parameters constrained
Δρ_max_ = 0.34 e Å^−3^
Δρ_min_ = −0.20 e Å^−3^

### 

Data collection: *APEX2* (Bruker, 2007 ▶); cell refinement: *SAINT* (Bruker, 2007 ▶); data reduction: *SAINT*; program(s) used to solve structure: *SHELXS97* (Sheldrick, 2008 ▶); program(s) used to refine structure: *SHELXL97* (Sheldrick, 2008 ▶); molecular graphics: *SHELXTL* (Sheldrick, 2008 ▶); software used to prepare material for publication: *SHELXTL*, *OLEX2* (Dolomanov, *et al.*, 2009 ▶) and *Mercury* (Macrae *et al.*, 2006 ▶).

## Supplementary Material

Crystal structure: contains datablock(s) I, global. DOI: 10.1107/S1600536813019636/ld2110sup1.cif

Structure factors: contains datablock(s) I. DOI: 10.1107/S1600536813019636/ld2110Isup2.hkl

Supplementary material file. DOI: 10.1107/S1600536813019636/ld2110Isup3.cml

Additional supplementary materials:  crystallographic information; 3D view; checkCIF report

## Figures and Tables

**Fig. 1.**
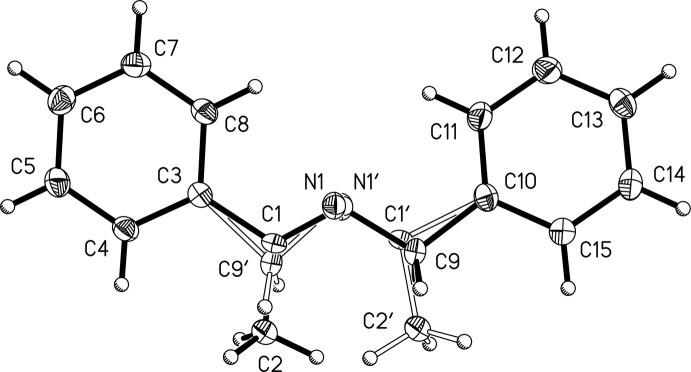
A view of the title compound with the atom numbering scheme. Displacement ellipsoids are shown at the 50% probability level. Primed atoms represent the disordered fraction of the CH2N=C(CH3) benzylidene portion of the molecule.

## supplementary crystallographic information

### Comment

Schiff base imines may be prepared by condensation of aldehydes and ketones with amines. The synthesis of the title compound, also known as (*E*)-*N*-(α-methylbenzylidene)benzylamine, may be accomplished by refluxing acetophenone and benzylamine in toluene using a Dean-Stark apparatus to separate the byproduct water. Recrystallizaiton by slow evaporation of a hexanes solution yields crystals of the *E*-isomer of the imine (Fig 1), with imine C=N bond length in the major component of the disorder of 1.292 (2) Å. The molecule has a mostly planar geometry with the benzyl group twisting slightly out of the plane, as indicated by the torsional angle N1—C9—C10—C11 of 8.5 (3)°. The herringbone molecular packing does not exhibit any significant face-to-face or edge-to-face π-stacking interactions.

The imine C=N bond in the title compound, 1.292 (2) Å, is comparable to that found in similar imines: [(*E*)-1-(Naphthalen-2-yl)ethylidene](naphthalen-1-ylmethyl)amine, 1.2650 (19) Å (Bruno *et al.*, 2012), 2-(*N*-benzyl-α-iminoethyl)phenol, 1.286 (2) Å (Filarowski *et al.* 1999), and (*S*)-(+)-*N*-(1-phenylethyl) salicylideneamine, 1.264 (4) Å (Liu *et al.*, 1997).

### Experimental

The title compound was obtained using a hybrid procedure of that reported in the literature (Guthrie *et al.*, 1973; Willoughby & Buchwald, 1994). A zinc catalyst was first prepared by adding 0.500 ml benzylamine to a mixture of 4 g of saturated ZnCl_2_ solution and 2 ml of ethanol. The solid catalyst was filtered out with a fritted funnel.

The catalyst was added to a mixture of 23.5 ml (0.200 mol) acetophenone and 21.8 ml (0.200 mol) of benzylamine in 100 ml of toluene. A reflux was conducted in a mineral oil bath, under argon with a Dean-Stark apparatus for 22 hrs at 120°C. After cooling, the solution was filtered through Celite and the toluene pumped off using a vacuum line.

The reflux product was then purified by distillation under vacuum (65 mTorr). Distillate collected at a distillation flask temperature of 109°C and vapor temperature of 45°C contained no imine product and was discarded. Distillate collected at a distillation flask temperature of ~190°C and vapor temperature of 80°C contained imine product.

To crystallize the final product, 50 ml of pentane was added to the second distillate and pumped off under vacuum line. This process was repeated 3 times. Melting point 42–43.5°C (Lit. 44–46 (Guthrie *et al.*, 1973)).

Recrystallization by slow evaporation of a hexane solution produced X-ray quality crystals.

### Refinement

All non-hydrogen atoms were refined anisotropically. Hydrogen atoms on carbon were included in calculated positions and refined using a riding model at C–H = 0.95, 0.98 and 0.99 Å and *U*_iso_(H) = 1.2, 1.5 and 1.2 × *U*_eq_(C) of the aryl, methyl and methylene C-atoms, respectively. The positions of the methyl H atoms were rotationally optimized. The disorder was modeled and refined with the help of similarity restraints on displacement parameters (SIMU), rigid bond restraints on 1–2 and 1–3 distances and anisotropic displacement parameters (DELU), and constraints on anisotropic displacement paramenters of the minor component (EADP). The extinction parameter (EXTI) refined to zero and was removed from the refinement.

### Crystal data

**Table:**

C_15_H_15_N	*F*(000) = 448
*M**_r_* = 209.28	*D*_x_ = 1.216 Mg m^−^^3^
Monoclinic, *P*2_1_/*n*	Mo *K*α radiation, λ = 0.71073 Å
Hall symbol: -P 2yn	Cell parameters from 5923 reflections
*a* = 5.4633 (4) Å	θ = 2.2–30.5°
*b* = 10.4173 (8) Å	µ = 0.07 mm^−^^1^
*c* = 20.2426 (15) Å	*T* = 125 K
β = 97.308 (1)°	Plate, colourless
*V* = 1142.71 (15) Å^3^	0.32 × 0.21 × 0.03 mm
*Z* = 4	

### Data collection

**Table:**

Bruker APEXII CCD diffractometer	3496 independent reflections
Radiation source: fine-focus sealed tube	2509 reflections with *I* > 2σ(*I*)
Graphite monochromator	*R*_int_ = 0.042
φ and ω scans	θ_max_ = 30.5°, θ_min_ = 2.0°
Absorption correction: empirical (using intensity measurements) (*SADABS*; Bruker 2007)	*h* = −7→7
*T*_min_ = 0.978, *T*_max_ = 0.998	*k* = −14→14
18404 measured reflections	*l* = −28→28

### Refinement

**Table:**

Refinement on *F*^2^	Primary atom site location: structure-invariant direct methods
Least-squares matrix: full	Secondary atom site location: difference Fourier map
*R*[*F*^2^ > 2σ(*F*^2^)] = 0.047	Hydrogen site location: inferred from neighbouring sites
*wR*(*F*^2^) = 0.127	H-atom parameters constrained
*S* = 1.03	*w* = 1/[σ^2^(*F*_o_^2^) + (0.0568*P*)^2^ + 0.3065*P*] where *P* = (*F*_o_^2^ + 2*F*_c_^2^)/3
3496 reflections	(Δ/σ)_max_ < 0.001
160 parameters	Δρ_max_ = 0.34 e Å^−^^3^
105 restraints	Δρ_min_ = −0.20 e Å^−^^3^

### Special details

**Table:**

Geometry. All e.s.d.'s (except the e.s.d. in the dihedral angle between two l.s. planes) are estimated using the full covariance matrix. The cell e.s.d.'s are taken into account individually in the estimation of e.s.d.'s in distances, angles and torsion angles; correlations between e.s.d.'s in cell parameters are only used when they are defined by crystal symmetry. An approximate (isotropic) treatment of cell e.s.d.'s is used for estimating e.s.d.'s involving l.s. planes.
Refinement. Refinement of *F*^2^ against ALL reflections. The weighted *R*-factor *wR* and goodness of fit *S* are based on *F*^2^, conventional *R*-factors *R* are based on *F*, with *F* set to zero for negative *F*^2^. The threshold expression of *F*^2^ > σ(*F*^2^) is used only for calculating *R*-factors(gt) *etc*. and is not relevant to the choice of reflections for refinement. *R*-factors based on *F*^2^ are statistically about twice as large as those based on *F*, and *R*- factors based on ALL data will be even larger.

### Fractional atomic coordinates and isotropic or equivalent isotropic displacement parameters (Å^2^)

**Table:**

	*x*	*y*	*z*	*U*_iso_*/*U*_eq_	Occ. (<1)
N1	0.6967 (5)	0.9477 (3)	0.15570 (10)	0.0197 (4)	0.909 (2)
C1	0.5051 (3)	0.95089 (15)	0.11114 (9)	0.0153 (3)	0.909 (2)
C2	0.2936 (2)	1.04626 (13)	0.11017 (6)	0.0223 (3)	0.909 (2)
H2A	0.3278	1.1058	0.1477	0.033*	0.909 (2)
H2B	0.1399	0.9998	0.1139	0.033*	0.909 (2)
H2C	0.2769	1.0946	0.0683	0.033*	0.909 (2)
C9	0.7279 (4)	1.04393 (17)	0.20835 (9)	0.0211 (3)	0.909 (2)
H9A	0.5803	1.0444	0.2321	0.025*	0.909 (2)
H9B	0.7430	1.1299	0.1884	0.025*	0.909 (2)
N1'	0.704 (6)	0.952 (4)	0.1458 (14)	0.0197 (4)	0.091 (2)
C1'	0.724 (4)	1.0191 (18)	0.1996 (11)	0.0153 (3)	0.091 (2)
C2'	0.582 (2)	1.1406 (12)	0.2104 (6)	0.0223 (3)	0.091 (2)
H2'A	0.4768	1.1632	0.1691	0.033*	0.091 (2)
H2'B	0.6975	1.2107	0.2232	0.033*	0.091 (2)
H2'C	0.4782	1.1263	0.2459	0.033*	0.091 (2)
C9'	0.458 (4)	0.972 (2)	0.1105 (11)	0.0211 (3)	0.091 (2)
H9'A	0.4371	1.0570	0.0889	0.025*	0.091 (2)
H9'B	0.3250	0.9563	0.1386	0.025*	0.091 (2)
C3	0.48698 (19)	0.85340 (10)	0.05684 (5)	0.0171 (2)	
C4	0.2827 (2)	0.84705 (11)	0.00784 (6)	0.0206 (2)	
H4A	0.1521	0.9071	0.0084	0.025*	
C5	0.2681 (2)	0.75399 (12)	−0.04178 (6)	0.0236 (3)	
H5A	0.1277	0.7508	−0.0746	0.028*	
C6	0.4574 (2)	0.66577 (12)	−0.04357 (6)	0.0242 (3)	
H6A	0.4473	0.6022	−0.0775	0.029*	
C7	0.6625 (2)	0.67147 (12)	0.00486 (6)	0.0232 (2)	
H7A	0.7931	0.6115	0.0039	0.028*	
C8	0.6775 (2)	0.76402 (11)	0.05436 (6)	0.0205 (2)	
H8A	0.8185	0.7670	0.0870	0.025*	
C10	0.9543 (2)	1.01713 (11)	0.25738 (5)	0.0200 (2)	
C11	1.0927 (2)	0.90513 (12)	0.25703 (6)	0.0255 (3)	
H11A	1.0448	0.8404	0.2249	0.031*	
C12	1.3012 (2)	0.88711 (13)	0.30342 (6)	0.0273 (3)	
H12A	1.3949	0.8104	0.3026	0.033*	
C13	1.3727 (2)	0.98068 (13)	0.35076 (6)	0.0250 (3)	
H13A	1.5162	0.9688	0.3819	0.030*	
C14	1.2336 (2)	1.09130 (12)	0.35225 (6)	0.0247 (3)	
H14A	1.2802	1.1550	0.3850	0.030*	
C15	1.0257 (2)	1.10962 (12)	0.30587 (6)	0.0226 (2)	
H15A	0.9313	1.1859	0.3072	0.027*	

### Atomic displacement parameters (Å^2^)

**Table:**

	*U*^11^	*U*^22^	*U*^33^	*U*^12^	*U*^13^	*U*^23^
N1	0.0192 (5)	0.0230 (6)	0.0165 (10)	−0.0001 (4)	0.0008 (6)	−0.0021 (8)
C1	0.0131 (7)	0.0150 (7)	0.0179 (5)	0.0033 (5)	0.0026 (5)	0.0024 (5)
C2	0.0197 (6)	0.0241 (6)	0.0223 (6)	0.0041 (5)	−0.0006 (5)	−0.0018 (5)
C9	0.0225 (6)	0.0220 (9)	0.0177 (8)	0.0008 (7)	−0.0018 (5)	−0.0052 (5)
N1'	0.0192 (5)	0.0230 (6)	0.0165 (10)	−0.0001 (4)	0.0008 (6)	−0.0021 (8)
C1'	0.0131 (7)	0.0150 (7)	0.0179 (5)	0.0033 (5)	0.0026 (5)	0.0024 (5)
C2'	0.0197 (6)	0.0241 (6)	0.0223 (6)	0.0041 (5)	−0.0006 (5)	−0.0018 (5)
C9'	0.0225 (6)	0.0220 (9)	0.0177 (8)	0.0008 (7)	−0.0018 (5)	−0.0052 (5)
C3	0.0162 (5)	0.0182 (5)	0.0170 (5)	−0.0013 (4)	0.0023 (4)	0.0022 (4)
C4	0.0167 (5)	0.0208 (5)	0.0234 (6)	0.0011 (4)	−0.0004 (4)	0.0002 (4)
C5	0.0199 (5)	0.0256 (6)	0.0240 (6)	−0.0025 (5)	−0.0026 (4)	−0.0025 (5)
C6	0.0247 (6)	0.0224 (6)	0.0255 (6)	−0.0028 (5)	0.0029 (5)	−0.0042 (5)
C7	0.0207 (5)	0.0221 (6)	0.0269 (6)	0.0027 (4)	0.0039 (4)	−0.0006 (5)
C8	0.0164 (5)	0.0236 (6)	0.0212 (5)	0.0013 (4)	0.0014 (4)	0.0015 (4)
C10	0.0199 (5)	0.0231 (6)	0.0166 (5)	−0.0012 (4)	0.0008 (4)	0.0010 (4)
C11	0.0308 (6)	0.0240 (6)	0.0201 (6)	0.0020 (5)	−0.0021 (5)	−0.0035 (5)
C12	0.0309 (6)	0.0263 (6)	0.0235 (6)	0.0079 (5)	−0.0014 (5)	0.0007 (5)
C13	0.0231 (6)	0.0305 (6)	0.0201 (6)	0.0010 (5)	−0.0016 (4)	0.0029 (5)
C14	0.0248 (6)	0.0261 (6)	0.0222 (6)	−0.0039 (5)	−0.0008 (5)	−0.0044 (5)
C15	0.0223 (6)	0.0221 (6)	0.0227 (6)	0.0006 (4)	0.0008 (4)	−0.0016 (4)

### Geometric parameters (Å, º)

**Table:**

N1—C1	1.292 (2)	C3—C8	1.4020 (15)
N1—C9	1.457 (3)	C4—C5	1.3908 (16)
C1—C3	1.490 (2)	C4—H4A	0.9500
C1—C2	1.5224 (17)	C5—C6	1.3876 (17)
C2—H2A	0.9800	C5—H5A	0.9500
C2—H2B	0.9800	C6—C7	1.3932 (16)
C2—H2C	0.9800	C6—H6A	0.9500
C9—C10	1.510 (2)	C7—C8	1.3855 (16)
C9—H9A	0.9900	C7—H7A	0.9500
C9—H9B	0.9900	C8—H8A	0.9500
N1'—C1'	1.289 (18)	C10—C11	1.3911 (17)
N1'—C9'	1.457 (19)	C10—C15	1.3952 (16)
C1'—C2'	1.516 (16)	C11—C12	1.3938 (17)
C1'—C10	1.60 (2)	C11—H11A	0.9500
C2'—H2'A	0.9800	C12—C13	1.3877 (17)
C2'—H2'B	0.9800	C12—H12A	0.9500
C2'—H2'C	0.9800	C13—C14	1.3828 (18)
C9'—C3	1.66 (2)	C13—H13A	0.9500
C9'—H9'A	0.9900	C14—C15	1.3920 (16)
C9'—H9'B	0.9900	C14—H14A	0.9500
C3—C4	1.3971 (15)	C15—H15A	0.9500
			
C1—N1—C9	120.1 (2)	C3—C4—H4A	119.6
N1—C1—C3	118.00 (15)	C6—C5—C4	120.40 (11)
N1—C1—C2	124.87 (17)	C6—C5—H5A	119.8
C3—C1—C2	117.12 (12)	C4—C5—H5A	119.8
N1—C9—C10	111.31 (15)	C5—C6—C7	119.27 (11)
N1—C9—H9A	109.4	C5—C6—H6A	120.4
C10—C9—H9A	109.4	C7—C6—H6A	120.4
N1—C9—H9B	109.4	C8—C7—C6	120.44 (11)
C10—C9—H9B	109.4	C8—C7—H7A	119.8
H9A—C9—H9B	108.0	C6—C7—H7A	119.8
C1'—N1'—C9'	108.1 (19)	C7—C8—C3	120.83 (10)
N1'—C1'—C2'	126 (2)	C7—C8—H8A	119.6
N1'—C1'—C10	125.7 (16)	C3—C8—H8A	119.6
C2'—C1'—C10	106.0 (11)	C11—C10—C15	118.64 (10)
C1'—C2'—H2'A	109.5	C11—C10—C9	123.52 (11)
C1'—C2'—H2'B	109.5	C15—C10—C9	117.84 (11)
H2'A—C2'—H2'B	109.5	C11—C10—C1'	112.5 (6)
C1'—C2'—H2'C	109.5	C15—C10—C1'	128.8 (6)
H2'A—C2'—H2'C	109.5	C10—C11—C12	120.53 (11)
H2'B—C2'—H2'C	109.5	C10—C11—H11A	119.7
N1'—C9'—C3	93.1 (14)	C12—C11—H11A	119.7
N1'—C9'—H9'A	113.1	C13—C12—C11	120.32 (12)
C3—C9'—H9'A	113.1	C13—C12—H12A	119.8
N1'—C9'—H9'B	113.1	C11—C12—H12A	119.8
C3—C9'—H9'B	113.1	C14—C13—C12	119.56 (11)
H9'A—C9'—H9'B	110.5	C14—C13—H13A	120.2
C4—C3—C8	118.20 (10)	C12—C13—H13A	120.2
C4—C3—C1	121.88 (10)	C13—C14—C15	120.21 (11)
C8—C3—C1	119.92 (10)	C13—C14—H14A	119.9
C4—C3—C9'	111.3 (6)	C15—C14—H14A	119.9
C8—C3—C9'	130.5 (6)	C14—C15—C10	120.74 (11)
C5—C4—C3	120.87 (11)	C14—C15—H15A	119.6
C5—C4—H4A	119.6	C10—C15—H15A	119.6
			
C9—N1—C1—C3	177.6 (2)	C4—C3—C8—C7	−0.39 (16)
C9—N1—C1—C2	−3.0 (4)	C1—C3—C8—C7	179.07 (12)
C1—N1—C9—C10	176.2 (3)	C9'—C3—C8—C7	179.4 (12)
C9'—N1'—C1'—C2'	−26 (5)	N1—C9—C10—C11	−8.5 (3)
C9'—N1'—C1'—C10	174 (2)	N1—C9—C10—C15	172.2 (2)
C1'—N1'—C9'—C3	−170 (3)	N1—C9—C10—C1'	−9 (5)
N1—C1—C3—C4	177.9 (2)	N1'—C1'—C10—C11	−22 (4)
C2—C1—C3—C4	−1.5 (2)	C2'—C1'—C10—C11	175.3 (10)
N1—C1—C3—C8	−1.6 (3)	N1'—C1'—C10—C15	159 (3)
C2—C1—C3—C8	179.08 (12)	C2'—C1'—C10—C15	−4 (2)
N1—C1—C3—C9'	180 (6)	N1'—C1'—C10—C9	158 (8)
C2—C1—C3—C9'	0 (5)	C2'—C1'—C10—C9	−5 (4)
N1'—C9'—C3—C4	−175 (2)	C15—C10—C11—C12	−1.24 (19)
N1'—C9'—C3—C8	5 (3)	C9—C10—C11—C12	179.41 (15)
N1'—C9'—C3—C1	7 (5)	C1'—C10—C11—C12	179.5 (10)
C8—C3—C4—C5	0.44 (17)	C10—C11—C12—C13	0.2 (2)
C1—C3—C4—C5	−179.01 (13)	C11—C12—C13—C14	0.92 (19)
C9'—C3—C4—C5	−179.4 (10)	C12—C13—C14—C15	−1.07 (19)
C3—C4—C5—C6	−0.23 (18)	C13—C14—C15—C10	0.05 (19)
C4—C5—C6—C7	−0.05 (18)	C11—C10—C15—C14	1.10 (18)
C5—C6—C7—C8	0.10 (18)	C9—C10—C15—C14	−179.51 (14)
C6—C7—C8—C3	0.12 (18)	C1'—C10—C15—C14	−179.8 (12)
